# How Should We Best Estimate the Mean Recency Duration for the BED Method?

**DOI:** 10.1371/journal.pone.0049661

**Published:** 2012-11-16

**Authors:** John Hargrove, Hayden Eastwood, Guy Mahiane, Cari van Schalkwyk

**Affiliations:** The South African Department of Science and Technology/National Research Foundation Centre of Excellence in Epidemiological Modelling and Analysis, University of Stellenbosch, Stellenbosch, South Africa; University of Ottawa, Canada

## Abstract

BED estimates of HIV incidence from cross-sectional surveys are obtained by restricting, to fixed time *T*, the period over which incidence is estimated. The appropriate mean recency duration (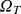) then refers to the time where BED optical density (OD) is less than a pre-set cut-off *C*, given the patient has been HIV positive for at most time *T*. Five methods, tested using data for postpartum women in Zimbabwe, provided similar estimates of 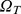 for *C* = 0.8: i) The ratio (*r*/*s*) of the number of BED-recent infections to all seroconversions over *T* = 365 days: 192 days [95% CI 168–216]. ii) Linear mixed modeling (LMM): 191 days [95% CI 174–208]. iii) Non-linear mixed modeling (NLMM): 196 days [95% CrI 188–204]. iv) Survival analysis (SA): 192 days [95% CI 168–216]. Graphical analysis: 193 days. NLMM estimates of 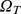 - based on a biologically more appropriate functional relationship than LMM – resulted in best fits to OD data, the smallest variance in estimates of 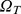, and best correspondence between BED and follow-up estimates of HIV incidence, for the same subjects over the same time period. SA and NLMM produced very similar estimates of 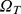 but the coefficient of variation of the former was >3 times as high. The *r*/*s* method requires uniformly distributed seroconversion events but is useful if data are available only from a single follow-up. The graphical method produces the most variable results, involves unsound methodology and should not be used to provide estimates of 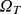. False-recent rates increased as a quadratic function of *C*: for incidence estimation *C* should thus be chosen as small as possible, consistent with an adequate resultant number of recent cases, and accurate estimation of 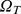. Inaccuracies in the estimation of 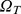 should not now provide an impediment to incidence estimation.

## Introduction

The BED Capture Enzyme Immuno-Assay (BED-CEIA or simply BED) measures the increasing proportion of anti-HIV-1 IgG in total IgG following HIV seroconversion [1]. HIV positive cases are classified as ‘recent’ seroconverters if they have a normalized optical density (OD) below a given cut-off on the BED assay. In principle the estimation of HIV incidence, *i*.*e*., the rate of occurrence of new infections, is a straightforward process using such a test, involving only enumerating the recent infections in a cross-sectional survey.

In practice, however, application of the BED method has resulted in over-estimates of HIV incidence [2]. Part of the problem with the application of the method is that there is no general agreement on how best to define the total times that patients spend in the recent state during their lives, let alone how best to estimate their mean value [3], [4].

The situation has clarified recently, however, with the demonstration that it is neither necessary nor desirable to estimate the mean recency duration over the whole life of a patient [5], [6]. Instead we should estimate the mean time spent in the recent state, *i*.*e*., the mean recency duration, during the time that patients have been HIV positive for at most some pre-defined time *T*. In this paper we investigate a number of approaches to the estimation of the mean recency duration for the BED method under this simplified scenario.

In so doing we investigate whether there is an optimum way of estimating the mean recency duration or whether several estimating procedures provide similar answers and whether, then, simple approaches will provide adequate answers. We also ask how estimates of the mean recency and incidence are affected by our choice of cut-off and whether these effects differ with our choice of estimation method.

Since all of the methods investigated below have been used previously in the literature, we do not in general attempt to provide formal statistical justification for their use, except where we have suggested modifications to the methods. Instead we contrast the resulting estimates in terms of their means and variances under different sets of input conditions, and then discuss under what conditions there could be reasons for preferring some estimators over others.

## Methods

### Data

Mean recency duration was estimated from data produced during the Zimbabwe Vitamin A for Mothers and Babies (ZVITAMBO) Trial, in Harare, Zimbabwe. All details regarding the study design, data collection and ethical clearance have been described previously [2], [5], [7].

**Table 1:** The numbers of independent BED tests provided by the 353 women who seroconverted during follow-up in the ZVITAMBO Trial, when either no exclusion criteria were applied, or where case were excluded if either there was only *s* = 1 sample per case, or the maximum time (*t* ^max^) between the last negative and first positive HIV tests was greater than 120 days.

	Inclusion criterion	
BED samples per case	All	*t^max^* ≤120 days s>1
1	167	–
2	89	28
3	35	25
4	21	17
5	24	17
6	8	5
7	8	7
8	1	1
Total	353	100

Briefly, between October 1997 and January 2000, 14,110 women and their babies were recruited within 96 hours of giving birth, tested for HIV at recruitment and at follow-up visits at 6-weeks, and 3, 6, 9, 12 …. 24-months. All available HIV positive samples from seroconverting mothers and from mothers who tested HIV positive at baseline, or at the 12-month visit, were tested by BED: subsets of these data were used to estimate mean recency duration. The time distribution of seroconversions during the first 12-months postpartum was also used to estimate HIV incidence [8], [9].

**Figure 1:**
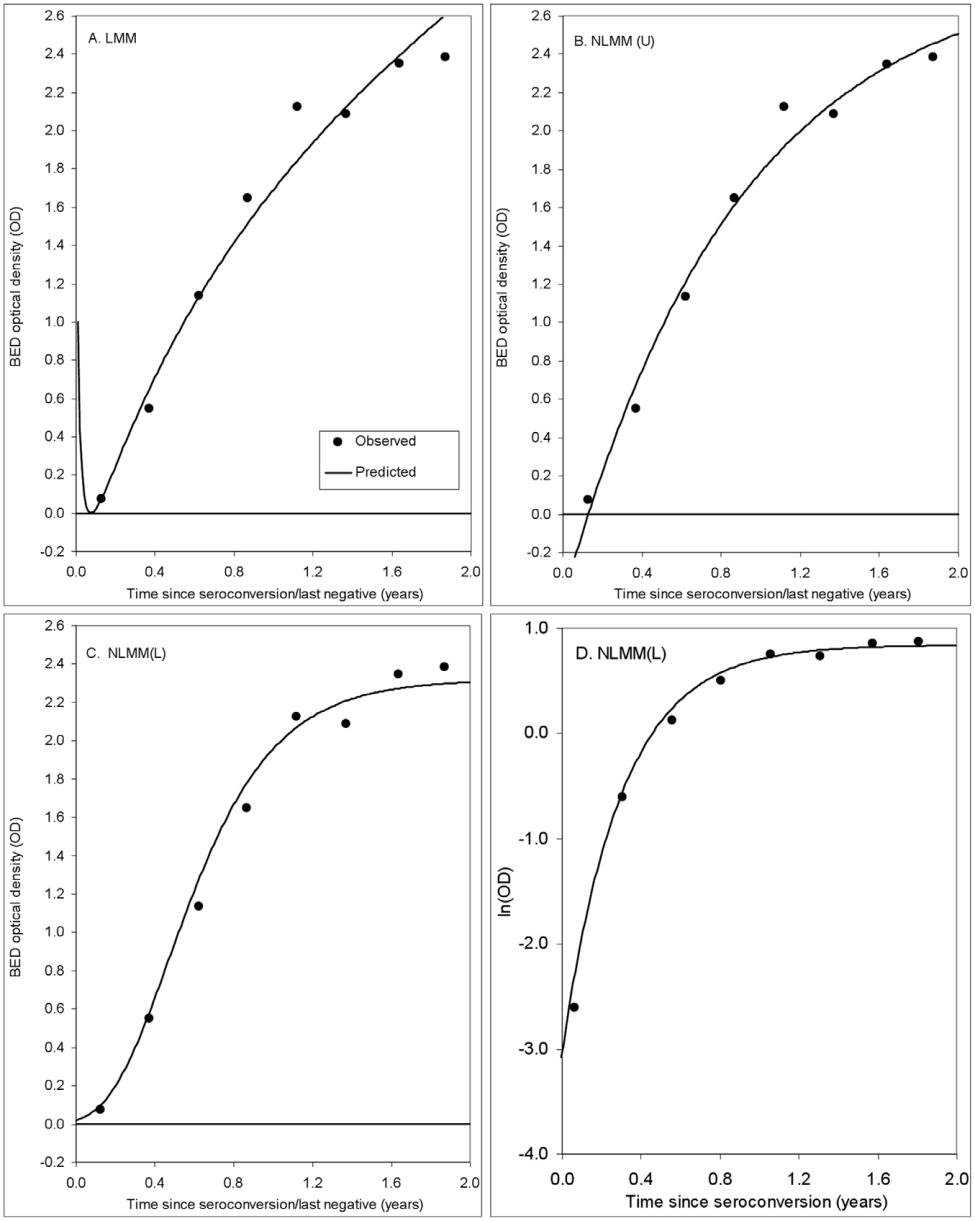
Fits to the BED OD data for a single seroconverting mother from the ZVITAMBO Trial. Predicted values obtained from: A. LMM. Linear regression of the square root of OD values against log time (*t*) since the last HIV negative test (Equation (7)). B. NLMM (U). Fitting the non-linear function given by Equation (8) to the untransformed BED OD data. C. NLMM (L). Fitting the non-linear function given by Equation (9) to the log-transformed BED OD data. D. Using the fit described in C, but now plotting log_e_(OD) on the ordinate.

### Incidence Estimation Using the BED

For BED data obtained from the analysis of cross-sectional survey data, two independent derivations [5], [6] suggest that a weighted average of the incidence rate over some pre-defined time *T* is best estimated by:
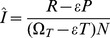
(1)


**Figure 2:**
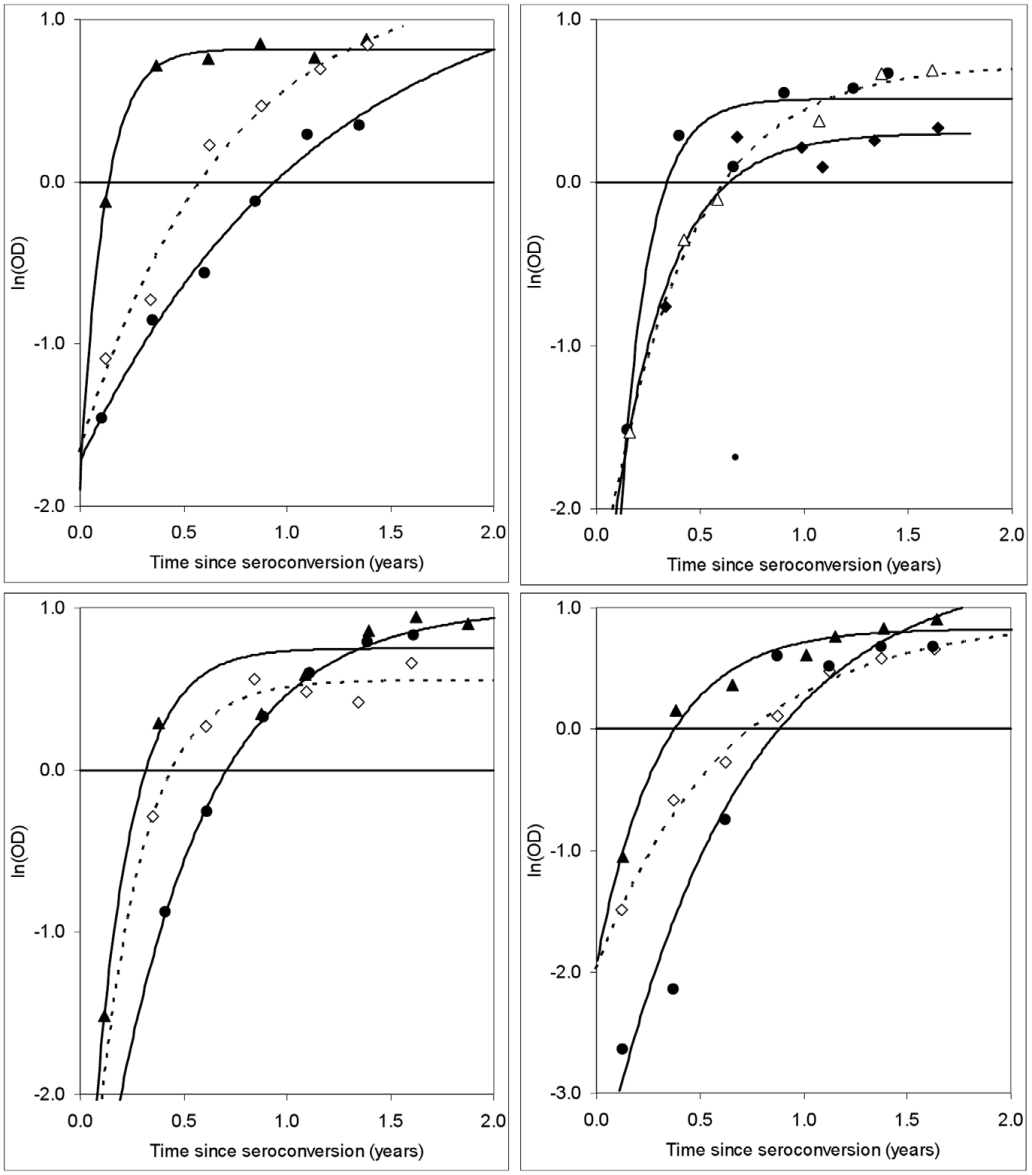
Fits to the BED OD data for ZVITAMBO mothers providing at least six BED samples following seroconversion. Fitting the non-linear function given by Equation (9) to the log-transformed BED OD data for 12 different women in the ZVITAMBO Trial who provided either six or seven separate BED results following seroconversion, and where the time between last negative and first positive HIV tests was at most 120 days. Plots of log_e_(OD) against estimated time since seroconversion.

where 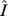 has units *T^−1^*, *P* and *N* are the numbers of seropositive and seronegative clients in the sample taken at time *T*, *R* the number of infections classified as recent, ε is the probability that a case tests as a recent infection given that the case has been HIV positive for time >*T*, and 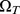 is the mean recency duration for those cases that have been alive and testing recent by BED while HIV positive for time ≤ *T*: 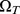 has the same units as *T*. *R*, Ω*_T_* and ε are functions of the pre-set OD cut-off (*C*). The variance of 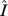 takes account of uncertainties in the estimates of both 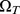
and ε, as detailed in [6]. When ε = 0 or, equivalently, when no adjustment is made for ε:
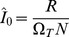
(2)


### Estimating the Mean Recency Duration

For pre-defined time *T* and cut-off *C*, set at levels convenient to the experimenter, we wish to estimate the mean time (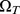) that a case spends in the recently-infected state (*i*.*e*., with BED optical density<*C*) while alive and infected for at most time *T*: formally 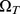 may be termed a *restricted mean survival time* with 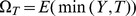, where *Y* is the time to crossing *C*. Without loss of generality, we take *T* as one unit of time, specifically one year for the ZVITAMBO study. Values of both 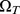 and ε are required in order to estimate the incidence from BED data obtained from cross-sectional surveys. The value of ε can be estimated in a given situation from a sample of cases known to be HIV positive for time >*T*. Estimates of 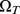 were obtained using five different methods:

**Figure 3:**
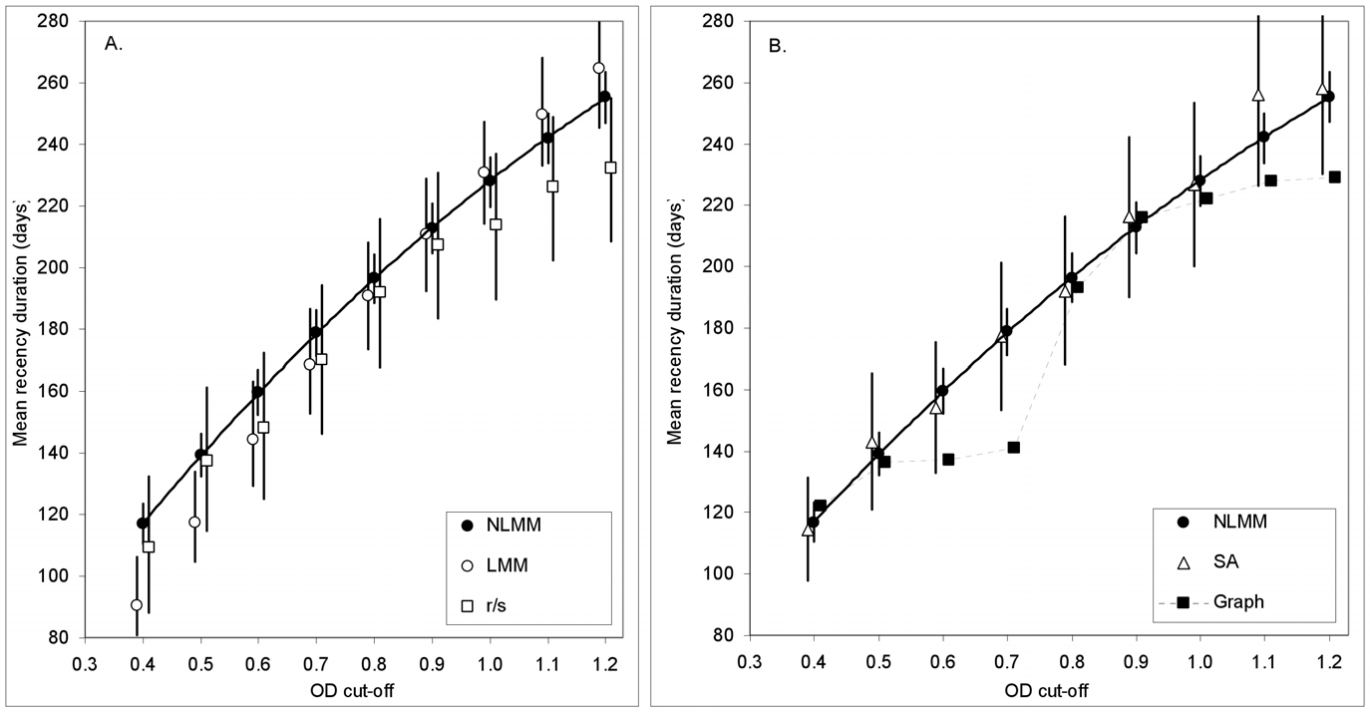
Mean recency durations, estimated using different methods, as a function of the BED pre-set optical density cut-off (*C*). Mean recency durations (with 95% confidence intervals) estimated using: A. Non-linear mixed modeling (NLMM); linear mixed modeling (LMM); the proportion of recent infections among seroconverters tested at one year postpartum (*r*/*s*). B. Survival analysis (SA); graphical analysis (Graph). NLMM estimates, included in both A and B as a reference, increased quadratically with *C*: OD = −64.4*C* ^2^+275.3*C*+17.4 (*R* ^2^>0.999). The dotted line indicates the greater variability inherent in the graphical method of estimation.

**Figure 4:**
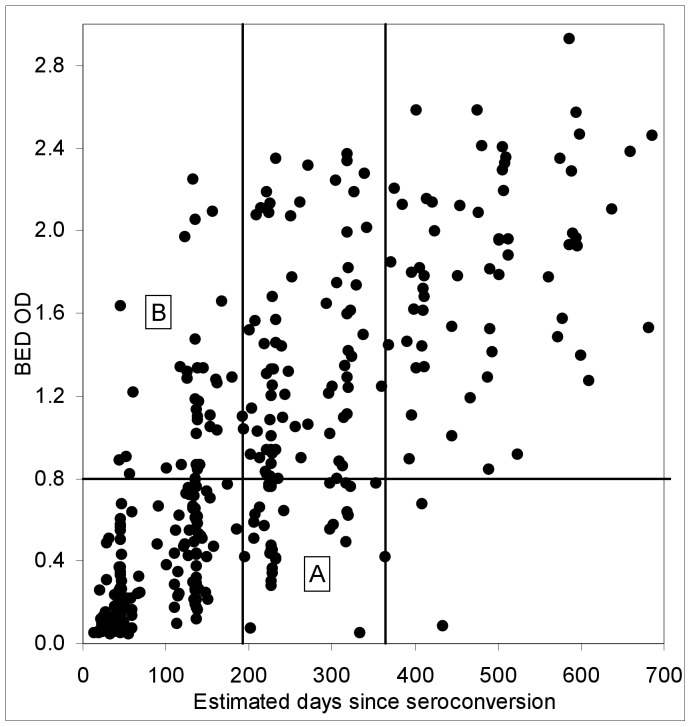
Graphical approach for estimating the mean recency duration. The graph shows a scatter plot of all BED OD values obtained from seroconverting women from the ZVITAMBO study, where the time between the last negative and first positive HIV tests did not exceed 120 days and where the woman provided at least four HIV positive samples. Horizontal line marks a pre-set OD cut-off of 0.8; vertical lines mark a pre-set cut-off of *T* = 365-days and a line whose position can be varied until the number of points in rectangles A and B are the same. Points in the other four rectangles are not used in this estimating procedure.

#### i) Proportion of recent infections among seroconverters, r/s [6]

For cases that are HIV negative at time 0 and tested again at time *T*, 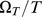 gives the probability that a seroconverter tests recent by BED – assuming a uniform distribution of seroconversion events over [0,*T*] [6]. It follows that, if *s* is the number of HIV positive cases observed at time *T*, among those HIV negative at time 0, and *r* is the number of these seroconverters that test recent by BED, given an OD cut-off of *C*:
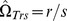
(3)


**Table 2:** Mean recency duration for seroconverting postpartum women in the ZVITAMBO Trial, estimated using five different approaches.

Method	Mean recency duration(95% CI) (days)	Coefficient of variation (%)
i. NLMM	196 (188–204)	2.0
ii. LMM	191 (174–208)	4.7
iii. Survival analysis	192 (168–216)	6.4
iv. Ratio *r*/*s*	192 (168–216)	6.4
v. Graphical	193	–

The optical density cut-off was fixed at 0.8 for all methods, minimum of two samples per case were required and the maximum allowable time between the last negative and first positive HIV tests was 120 days.

Notice that *r* here is the number of women testing recent at time *T* only among women who were HIV negative at baseline, whereas, more normally as in Equations (1) and (2), *R* refers to the number testing recent in a population cross sectional survey.

**Table 3:** Mean recency duration for seroconverting postpartum women in the ZVITAMBO Trial, estimated using non-linear mixed modeling (NLMM), as a function of the minimum number of samples (NS) allowable per client and the maximum period (*t_0_*) allowed between the last negative, and first positive, HIV tests; *n* denotes the resulting number of clients included in the test.

*t*_0_ (days)	NS	*n*	Mean recency (95% CrI) (days)	CoV(%)
80	2	32	176 (165–187)	3.2
80	3	27	179 (166–191)	3.5
80	4	23	193 (179–207)	3.7
120	2	100	196 (188–204)	2.0
120	3	71	199 (191–208)	2.2
120	4	47	194 (183–205)	2.9
160	2	109	193 (185–200)	2.0
160	3	78	196 (188–204)	2.1
160	4	51	192 (182–202)	2.6

The optical density cut-off was fixed at 0.8 for all estimates. The coefficient of variation (CoV) is defined as the standard error of the estimate divided by the mean, expressed as a percentage.

It has been argued that a good estimate for the mean recency duration will ensure equality between BED and follow-up estimates of incidence (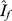) if both estimates are made over an identical period (*T*), using the same subjects [5]. Thus, taking 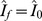, substituting in (2) and re-arranging, we get the mean recency duration, given a follow-up incidence rate of 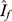:
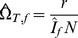
(4)


with variance given by:
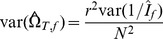
(5)where an approximation to 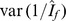 is derived using the delta method.
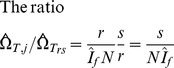
(6)is again independent of *r* and thus of *C* and has a numerical value close to one. The ratio *r*/*s* in (3) should thus provide an estimate of the mean recency duration that is close to the estimate that would be required to ensure equality between follow-up and BED estimates of incidence.

**Figure 5:**
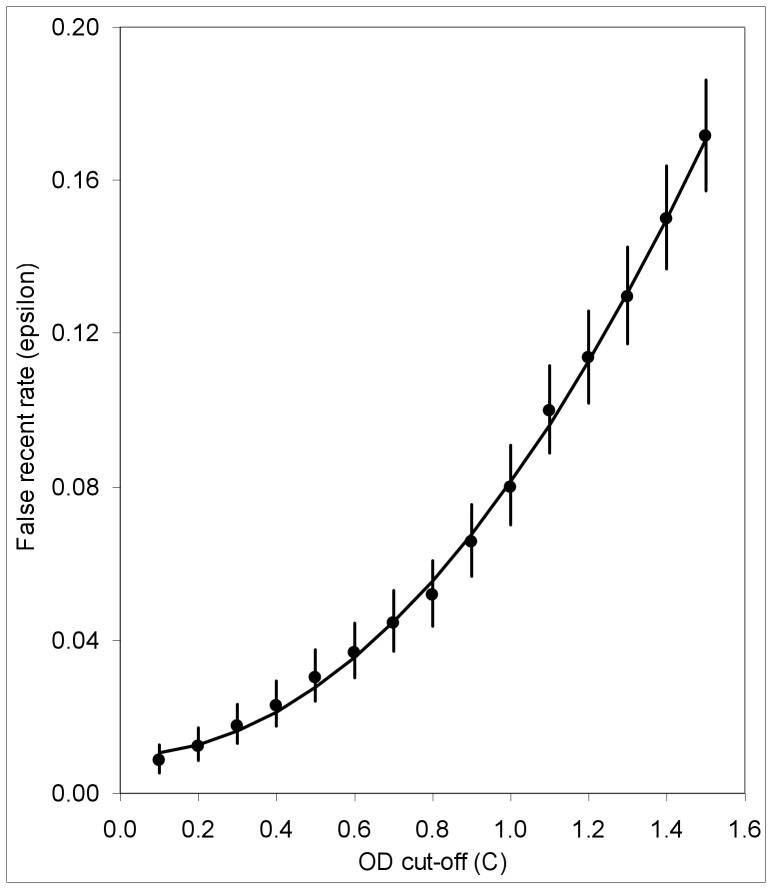
The relationship between the long-term false-recent rate (ε) and the pre-set optical density cut-off (*C*) in the ZVITAMBO Trial. The value of ε was estimated as the proportion of cases with a BED OD<*C* among women tested at *T* = 12-months postpartum, given that they had previously provided a positive HIV test at baseline. Error bars indicate the 95% confidence intervals.

**Figure 6:**
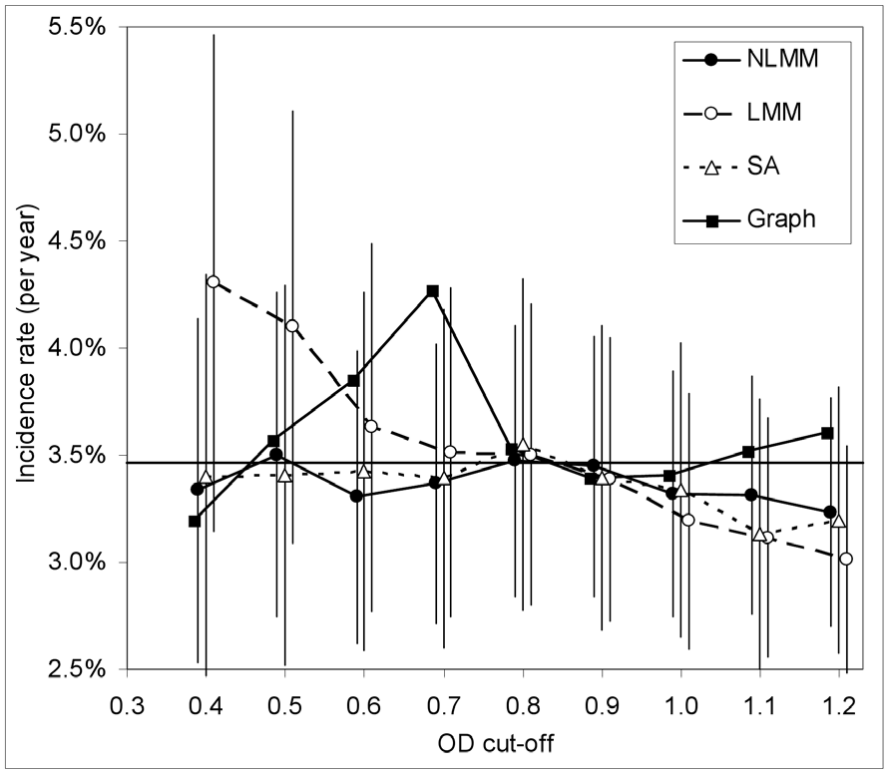
HIV incidence, estimated using BED, with the mean recency duration estimated using four different methods. HIV incidence (with 95% confidence intervals) among women during their first year postpartum in the ZVITAMBO Trial, calculated using estimates of the mean recency duration from non-linear mixed modeling (NLMM), linear mixed modeling (LMM), survival analysis (SA) and graphical analysis (Graph).

#### ii) Linear mixed model (LMM) [1], [2]

Transformation of the unbalanced longitudinal data produces a linear mean structure and allows, by solving for *t*, the estimation of the time (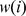 for case *i*) between the time at which OD begins to increase above baseline, and the time it reaches the OD cut-off (*C*) [1], [2]. Changing optical density for each individual *i* is modeled as:
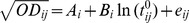
(7)


**Figure 7:**
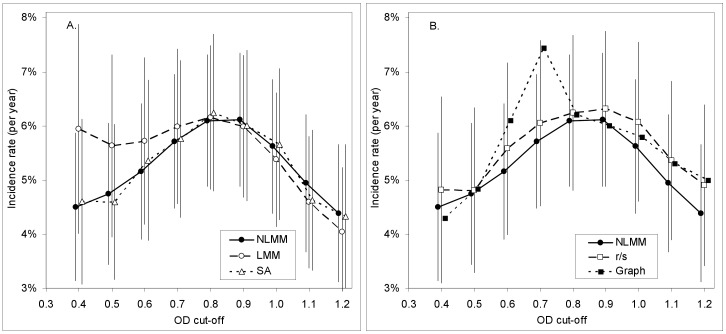
HIV incidence, estimated using BED, with the mean recency duration estimated using five different methods. HIV incidence (with 95% confidence intervals) in women during the period prior to their recruitment into the ZVITAMBO Trial. A. Non-linear mixed modeling (NLMM); linear mixed modeling (LMM); survival analysis (SA). B. The proportion of recent infections among seroconverters tested at one year postpartum (*r*/*s*); graphical analysis (Graph). Results for NLMM included for comparison.

where *A_i_* and *B_i_* are constants containing fixed and random effects, *t* ^0^ *_ij_* is the time at observation *j* since the last HIV negative test and the *e_ij_* are independent and identically distributed normal errors. The LMM approach to fitting these data studied changes over time within subjects and for the entire group. Each recency duration is defined as the time spent in the recent state, with the upper limit restricted to *T*. Bootstrap techniques were applied to these individual estimates to obtain the mean and confidence intervals for the mean recency duration 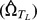, with the provisos noted above.

**Figure 8:**
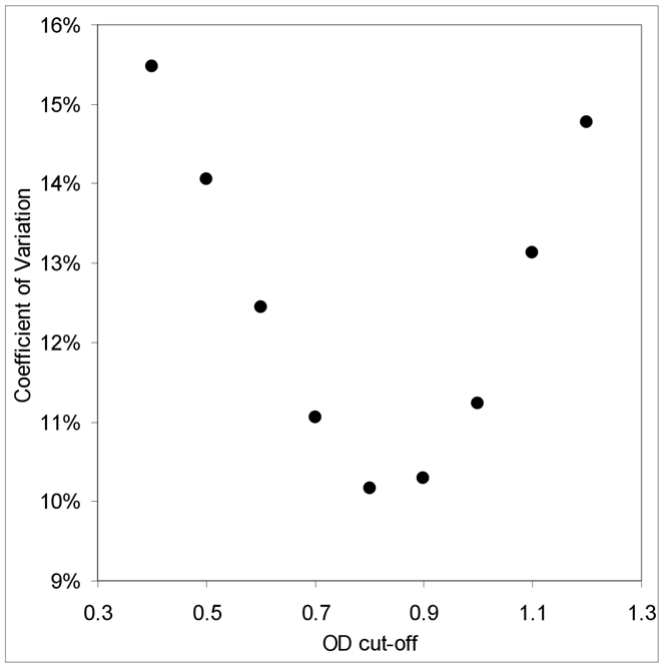
Variability in BED HIV incidence estimates as a function of optical density cut-off (*C*). The coefficient of variation for BED HIV incidence estimates obtained using the ZVITAMBO baseline data, as a function of the pre-set optical density cut-off (*C*). Incidence calculated using Equation (1) with values of 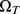 estimated by NLMM.

#### iii) Non-linear mixed model

(NLMM) [10]. Sweeting *et al*. [10] modeled changing BED optical density for each individual *i*, at observation *j*, as:
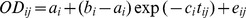
(8)


**Figure 9:**
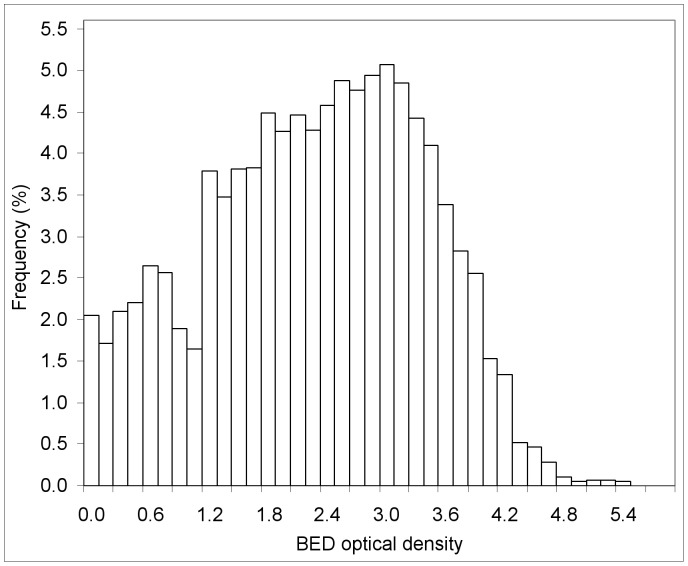
Distribution of BED optical density among women at recruitment into the ZVITAMBO Trial.

where *a_i_*, *b_i_* and *c_i_* are constants, *t_ij_* is the time since seroconversion and the *e_ij_* are independent and identically distributed normal errors. The date of seroconversion is assumed to be uniformly distributed between the dates of last negative and first positive HIV tests. Individual recency durations were obtained by using an inverse prediction technique [10]. A Bayesian approach using Markov Chain Monte Carlo (MCMC) methods is implemented to estimate the posterior distribution of the recency duration for each individual, with the upper limit restricted to *T*. Similarly, the distribution of the mean recency duration is obtained using the MCMC iterations. The mean recency duration 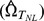 and credibility interval are obtained from this distribution. This method assumes that the underlying biomarker process increases monotonically. Due to measurement error, the observed measurements will fluctuate around the underlying trajectory and will not increase monotonically.

We investigated a variant of this function:
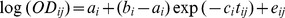
(9)


where *c_i_*>0, *a_i_*>*b_i_* and the *e_ij_* are independent and identically distributed normal errors. This function also approaches an asymptote for large *t* and has the further property that it goes to zero as *t* → −∞.

#### iv) Survival analysis (SA) [9], [10]

Assuming no underlying parametric model for the recency duration, the SA approach is followed when recognizing the data as being double interval censored. The exact times of seroconversion and of reaching a pre-defined OD cut-off are not known, but intervals for each are obtained from the data. This creates an interval of the shortest and longest possible recency durations for each individual. Sweeting *et al*. [10] used such data to calculate the upper and lower bounds of the cumulative distribution function for the recency duration. They found this to be of little practical use and did not pursue the method to provide mean values of the recency duration.

They also noted that carrying out a univariate survival analysis of the double interval censored data, as if they were single interval censored, assumes an incorrect likelihood function. We consider an alternative approach where we approximate the time of seroconversion to be the mid-point between the times of the last negative and first positive HIV tests. Given that, for our data, the maximum time between these tests was set at 120 days (average 83 days), the average error in the assumed date of seroconversion should be small. The data are then single interval censored and Turnbull’s modification of the Product-Limit Estimator yields a survival function which, when integrated over [0,*T*], provides a mean recency duration 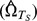
and corresponding confidence intervals. SA has the advantage of having no parametric assumptions, but the disadvantage that it does not use information on the shape of the increase in OD with *t*.

#### v) Graphical method [1]

For seroconverting cases that have been HIV positive for less than time *T*, the mean recency duration was estimated from a scatter plot of BED OD values and the time (*t_S_*) since seroconversion, estimated here as the mid-point between the last negative and first positive HIV tests. For a given choice of *C* we seek a recency duration 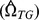 that produces an equal number of cases where: i) *t_S_*<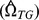 and OD≥*C* and ii) where 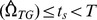 and OD<*C* – *i*.*e*., which results in equal values of the sensitivity and specificity over time *T* [3].

### Data Analysis

Data were analyzed using Microsoft Excel, R version 2.14.1 [11] and WinBUGS 14 [12]. The code used to produce the NLMM estimates was that used in [10].

## Results

### HIV and BED Test Results at Baseline and Follow-up

Of 14,110 women recruited, 9562, 4495 and 53 mothers tested HIV negative, positive and indeterminate, respectively. During follow-up, 353 of the initially HIV negative mothers were seen to seroconvert: the numbers of times that each of these cases was seen, and tested for HIV and for BED optical density (OD), are shown in Table 1.

At 12-months postpartum, 6829 of the baseline HIV negative cases were retested: 6595 still tested HIV negative and *s* = 234 tested positive. All of the HIV positive cases were tested using BED and *r* = 123 of them had an OD<0.8 – *i*.*e*., they tested “recent” at the commonly used OD cut-off. Of the baseline HIV positive cases, 3010 were seen again at 12-months and all were confirmed as still HIV positive. Of these cases, 2749 were tested using BED and 142 of them had an OD<0.8, *i*.*e*., tested “recent”, despite having been HIV positive for at least one year.

The *r*/*s* estimator used only the above HIV and BED test data from baseline and twelve months. The other four methods used all of the qualifying data available from follow-up. For mixed effects modeling and survival analysis a minimum of *S* = 2 samples per patient are required: there were 186 such cases (Table 1). To minimize uncertainty regarding the time of seroconversion it is, moreover, necessary to limit the maximum time (*t* ^max^) allowed between last negative and first positive samples [2]: initially *t* ^max^ was set at 120 days. The above selection criteria resulted in a sample of 100 women (Table 1), who were used to compare the performance of the LMM, NLMM, SA and graphical estimators. Sensitivity of the estimates of 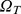 to these selection criteria was investigated by varying *S* between 2 and 4 and *t* ^max^ between 80 and 160 days. Estimates were obtained for OD cut-offs (*C*) ranging from 0.4 to 1.2.

### Estimates of the Mean Recency Duration

#### i) Estimates using r/s

Previously published survival analysis of the follow-up HIV test data provided an estimate of the probability (*J*) of seroconverting during the first year postpartum of 3.4% (95% CI: 3.0%–3.8%) or, equivalently, an *instantaneous incidence rate* (*I*) of 3.46% per year (95% CI: 3.05%–3.87%), approximating *I* from *I* = −ln(1−*J*), and using the approximation that the incidence is constant over the interval [0,*T*] [8], [9].

Of the cases testing HIV negative at baseline, and then tested again at 12-months, *N* = 6595 tested HIV negative. Seroconversion was detected among *s* = 234 of these cases, and *r* = 123 of these tested recent by BED when using a cut-off of *C* = 0.8. For this cut-off it is confirmed that the mean recency duration provided by Equations (3) and (4) are closely similar, as expected:
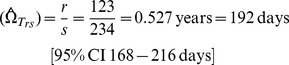


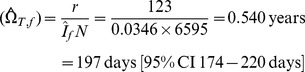



Accordingly, further reports will contrast only 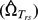 to the estimates provided by the regression, survival analysis and graphical approaches.

#### ii) & iii) Linear and non-linear mixed modeling

Figure 1 provides an illustration of the fits achieved using the functions in Equations (7), (8) and (9) to data for the single ZVITAMBO seroconverting woman who provided BED samples at eight separate time points post-seroconversion. While all of the functions provide reasonable fits to these data, Equation (7) predicts that OD → ∞ both as *t* → ∞, and as *t* → 0 (Figure 1A). Equation (8) matches biological observations better, in that the OD approaches a finite asymptote as *t* → ∞. However, OD → −∞ as *t* → −∞ so that the predicted value of the OD can be negative for small positive values of *t* (Figure 1B). This property could be avoided by insisting that *b_i_*>0, but the function still goes to −∞ as *t* → −∞ and has an inappropriate shape in the neighborhood of *t* = 0. Equation (9) has the property of approaching finite asymptotes for both large and small times: OD → exp(*a_i_*) as *t* → ∞, and OD → 0 as *t* → −∞. Unlike Equation (8) therefore, it never predicts negative values of OD and provides better fits to the data in the neighborhood of *t* = 0 (Figure 1C), matching the observation that there is a delay, following seroconversion, in the increase in BED optical density. Figure 1D shows the same fit as for Figure 1C but plotting log_e_(OD) on the ordinate.

The likely form of the increase of the BED optical density with time since seroconversion can only be well judged from results for those individuals who are seen a number of times over an extended period. Figure 1D and Figure 2 shows plots for all thirteen cases in the ZVITAMBO data set where seroconverting subjects provided at least six independent BED results, and where the time between last negative and first positive HIV tests was at most 120 days. All cases were followed up for more than a year and 8/13 followed for more than 18 months, and in all cases Equation (9) provided a good fit to the data for individual clients (Figure 2). For 33/47 (70%) of cases where there were at least four BED results, Equation (9) provided a better fit to the data than Equation (8): accordingly, results are only presented below for fits using Equation (9).

BED data for all qualifying seroconverting cases were analyzed using LMM and NLMM. For the NLMM method, estimates of 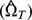 increased quadratically for *C* varying between 0.4 and 1.2. For *C*<0.6 the LMM estimates were significantly lower than those from the NLMM, but there were no significant differences between the estimates for *C* = 0.6–1.2 (Figure 3A). The *r*/*s* estimates did not differ significantly from either the LMM or NLMM estimates for C≤1.0, although the point estimates were consistently lower than the NLMM estimates and diverged increasingly from them for *C*>1.0. The major differences between the estimators lay in the coefficient of variation (CoV, *i*.*e*., the standard error divided by the mean), which was, on average, 2.3 and 3.2 times as high for the LMM and *r*/*s* estimates, respectively, as for the NLMM estimates.

#### iv) Survival analysis

For estimates at each *C*≤1.0 there were negligible differences (on average 1.9%) between the SA and NLMM estimates (Figure 3B). The average CoV for the SA estimate was, however, >3 times as high as for the NLMM estimates.

#### v) Graphical method

Figure 4 provides an example of the use of this method. For a given choice of the OD cut-off, *C*, the vertical line, shown at *t* = 193 days in Figure 4, is moved along the time axis until the numbers of points in rectangle A (those cases that have been infected for at least *t* days, but less than *T* = 365 days and test as recent infections, with BED OD<*C*) is the same as those in rectangle B (those cases that have been infected for a period less than *t*, but test as long-term infections with BED OD≥*C*).

Whereas it was not possible to provide confidence intervals for this simple method the estimates of 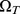 are markedly more variable than those provided by the LMM, NLMM and SA methods (Figure 3A, B). This variability, and the approximately step-wise increase in 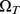 with increasing *C*, results from the regular timing of follow-up visits in the ZVITAMBO Trial, which produced approximate vertical alignment of many of the readings in Figure 4. This effect was particularly noticeable for times close to seroconversion.

At the commonly used cut-off of *C* = 0.8 the mean of the estimates from all five methods was 193 days (range 191–196 days), with all estimates differing by <2% from this figure and with an average deviation of 0.8%. None of the four estimates for which it was possible to provide 95% confidence intervals differed significantly from each other (Figure 3, Table 2). The CoV for the NLMM estimate was, however, less than a half of that for the LMM estimates and less than one third of that for the SA and *r*/*s* estimates (Table 2).

### Sensitivity of the Estimates of 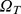 to Data Selection

Estimates of the mean recency duration were fairly insensitive to the way in which data were selected. When the minimum allowable number of samples per client was varied between 2 and 4, and *t* ^max^ between 80 and 160 days, the estimates of 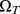
estimated using NLMM differed by at most 8% from the mean of the estimated values (Table 3).

### Variation of the False Recent Rate with the OD Cut-off C

Whereas we have, for completeness, examined the way in which various estimators perform over a large range of *C*, it is also clear that the long-term false-recent rate (ε) increases with *C*. In the case of the ZVITAMBO Trial, ε increases quadratically with changes in *C* between 0.1 and 1.5 (Figure 5). Given that a central aim of estimating procedures such as BED is to minimize the value of ε, it will make sense to use the lowest value of *C* consistent with other considerations (see below).

### Incidence Estimates as a Function of C and the Mean Recency Estimation Method

#### 1. Incidence over the first 12-months postpartum

BED data from the ZVITAMBO Trial, for women testing HIV positive both at baseline and at 12-months postpartum, were used to estimate ε. Data for these cases cannot therefore be used to obtain BED estimates of incidence over this period, since this incidence estimate, obtained from Equation (1), involves using the estimated value of ε.

BED estimates of the incidence over this period can, however, be obtained legitimately via Equation (2), which does not involve ε, as long as we use only the BED data for seroconverting women who tested HIV positive at 12-months postpartum. It is then possible to compare the follow-up estimate of incidence (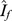), shown as a horizontal line in Figure 6, with estimates arising from Equation (2) for varying values of *C*, and the appropriate values of 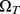, obtained using different estimators. Incidence estimates appropriate to the *r*/*s* estimate of 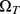
are not shown since these are, as evident from Equation (6), a constant multiple of 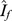.

For all values of *C* tested, the NLMM estimates of incidence showed the smallest deviation from the follow-up estimate of 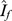 = 3.46%, varying only between 3.23% and 3.50%: the SA and NLMM estimates were closely similar for all *C* ≤1.0. For *C* = 0.8, 0.9 and 1.0 incidence estimates arising from the use of all methods differed from each other, and from 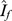, by amounts that were small compared to the size of the confidence intervals. For C<0.8 the LMM and graphical methods varied more substantially from NLMM and SA estimates (Figure 6).

Despite the differences in CoVs between the estimates of 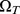 arising from the NLMM, LMM and SA methods, there was little difference between the sizes of the confidence intervals for the incidence estimates. The sizes of these latter intervals are thus dominated by counting errors arising from the use of the incidence estimator defined by Equation (2).

#### 2. Incidence over the year prior to birth

The way in which our choice of C, and thus of 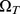 and ε, affects incidence estimates can also be tested using the ZVITAMBO baseline BED data since these data were not used in the estimation of either 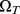 or ε. In this case, however, there is no follow-up incidence estimate for comparison, since baseline marked the first time that any of the ZVITAMBO subjects had been seen in the study.

The contrasts between estimating methods seen in the postpartum incidence estimates are, as expected, largely repeated for the baseline analysis (Figure 7) with the graphical method showing the greatest variability with changes in *C*, and with differences between the estimates from other methods all small by comparison with the size of the confidence intervals.

For the NLMM method, which produces estimates of 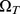 with the smallest CoVs, the CoVs of the resulting incidence estimates also change in a regular manner with increasing *C* and show a minimum value for *C*∼0.8 (Figure 8). That is to say that the value of *C* commonly used in the application of the BED method is also associated with the smallest coefficient of variation.

The consistent trend in the baseline HIV estimates with changes in *C* are different from those seen in the 12-months postpartum results (*cf* Figures 6 and 7) where the NLMM estimates of incidence were largely independent of *C.* The source of the variation in the baseline estimates appears to lie in the distribution of the baseline optical density data (Figure 9), which show a small local peak for *C* in the region 0.6–0.8, and a sharp increase in frequency at *C*≈1.2. Any particular choice of *C* results in the estimation of incidence over a particular time period prior to sampling and the distribution of BED optical density in Figure 9 suggests that incidence is not uniform over the period leading up to parturition.

## Discussion

### Which Estimator?

For *C* in the neighborhood of 0.8 there is little difference between the values of 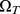 arising from any of the five estimators tested here. However, at all values of *C* tested, NLMM estimates of 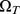 had markedly smaller variance than the LMM, SA and *r*/*s* estimates. As such the NLMM method provides the best available approach for estimating 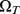, for data sets such as those from the ZVITAMBO study where there is sufficiently detailed information to support meaningful regression analysis. Only the NLMM and LMM methods use information on the changes in BED optical density with time since seroconversion and, accordingly, provide estimates with lower variance. The NLMM is markedly superior to the LMM in this regard, being based on a more appropriate functional relationship, with sensible limits for large and small times post-HIV infection. This ensures better fits to the data and markedly smaller variation than for the LMM. NLMM should accordingly always be used in preference to LMM.

Estimates of 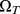 from the simple graphical method show the greatest variability and, as demonstrated above, are strongly dependent on the time distribution of the seroconversion events. There are, moreover, methodological objections to the use of the method: the data involve repeated measurements from a number of individuals, and each measurement is used to define a probability. The data are thus clearly correlated; for example if an individual’s data point is “false recent”, the next is also likely to be “false recent”. Moreover, measurements become less frequent with increasing time since last negative HIV test. For all of the above reasons, the graphical method should be used, if at all, only to provide first approximations of 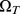.

The *r*/*s* method is dependent on the assumption of a uniform distribution of seroconversion events across the period [0,*T*] and, as is evident from Figure 9, there can sometimes be serious violations of this assumption. On the other hand, it is noteworthy that the baseline incidence estimates (Figure 7) from the *r*/*s* and the NLMM methods were closely similar and showed the same pattern of changes as *C* was varied between 0.4 and 1.2. Moreover, in situations where follow-up of individual seroconverting cases is not as extensive as in the ZVITAMBO Trial, the relative advantage of regression approaches for the estimation of 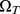
will be diminished and the *r*/*s* method would provide a reasonable alternative.

Our findings support previous work suggesting that the use of SA will be problematic for estimating 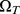 [10]. Even when we used an approximation, which under-estimates the variance of the estimate, the coefficient of variation for the SA estimates was markedly larger than that for the NLMM method. As with the *r*/*s* method, however, the relative advantage of the NLMM method will be reduced in the situation where there is limited follow-up of individual clients. We checked this by trimming the ZVITAMBO data to exclude, for each client, all but the first BED sample and the sample taken closest to 12-months postpartum. The SA estimate derived using these data differed by <2% from the estimate obtained using the full data set, and the coefficient of variation was only marginally larger. The NLMM method does not provide a meaningful confidence interval with these minimal data.

### Which Cut-off?

For values of the OD cut-off between *C* = 0.6 and 1.2 the BED estimates of incidence over the 12-months postpartum period, obtained using the NLMM estimates of 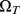, accord closely with the follow-up estimate of about 3.46% per year. These results provide strong support for this method of estimating 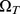, but do not suggest which cut-off should be preferred. This question is better answered by considering the BED estimates arising from the ZVITAMBO baseline data – which mirrors more closely the cross-sectional surveys encountered in practice, where there tends to be a preponderance of cases with long-term infections. It is then necessary to adjust the BED incidence estimates for the long-term false-recent cases, calculating incidence using Equation (1).

The choice of cut-off is then decided by a trade-off of the advantages of increasing *C* such that we observe a greater number of recent cases (*R*), and decreasing *C* such that we reduce the value of ε (Figure 5). The net result of such changes sees the CoV of the baseline incidence estimates showing a well-marked minimum (Figure 9) suggesting that there is no reason to change from the value of *C* = 0.8 currently in common use.

### Which Incidence Estimate?

Notwithstanding the results of the previous section, the NLMM estimates of baseline HIV incidence in Figure 9 vary between 4.5% and 6%, depending on the chosen value of *C*. In understanding the reasons for this variation it is important to remember that, as *C* is increased, HIV incidence is averaged over progressively longer periods. Moreover, two independent things are happening: i) 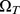 is changing, in a manner that is determined by the properties of the test, and is independent of the distribution of the BED OD values in the cross-sectional sample being analyzed: ii) The number of cases counted as recent is changing, at a rate which is a function of the pattern of seroconversions prior to the sampling time.

Thus, with reference to Figure 9, the rate of accumulation of cases classified as recent becomes progressively more rapid as *C* increases to 0.6, and then progressively less rapid for *C* increasing between 0.6 and 1.2. These changes are reflected in changes in HIV incidence with *C* (Figure 7), consistent with the idea that the rate of acquisition of new infections was not constant over the period prior to a woman’s enrollment in the ZVITAMBO Trial. This is unsurprising given that enrollment occurred within 96 hours of parturition, so all women were in the same synchronized physiological state and BED data are reflecting events during the preceding pregnancy. Indeed, since *C* = 1.2 corresponds to 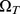
∼260 days (Figure 3), just short of the approximate mean duration of human gestation, the distribution of BED values for *C* between 0 and 1.2 basically reflects events during most of pregnancy.

The results in Figure 7 are consistent with the idea that acquisition of HIV infection is relatively low shortly before birth. It is not unreasonable to think that sexual activity, and thus the risk of HIV infection, is reduced at this late stage of pregnancy, compared with the middle stages of pregnancy. Similarly, the sudden jump in frequency at *C* = 1.2 coincides approximately with the time that the women became pregnant – which is the only time that we can be absolutely certain that they had all had unprotected sex and were thus at risk of HIV infection.

On the above interpretation, the results in Figure 8 reflect true changes in the pattern of HIV infection in the year prior to birth for women in the ZVITAMBO Trial. Similar situations are likely to occur in other sampling scenarios. Most women making a first antenatal clinic visit, for example, are likely to be at a similar stage of pregnancy, and seroconversion events in the year prior to the test may be expected to be distributed in a distinctly non-uniform way.

### Limitations

We caution that the present study is based on the application of various methods to a single set of data, all derived from postpartum women, from a single city in Zimbabwe and all infected with a single clade of HIV. The results apply, moreover, only to the BED method. Similar studies are needed to establish the extent to which our results can be generalized in other settings and using other bio-marker methods.

### Conclusion

The estimation of the mean recency duration for cases which have been HIV positive for some defined finite period *T* proves much less problematic than previous attempts to estimate the life-time mean recency duration [1], [2], [3]. The estimation of mean recency durations should not thus be seen as a major obstacle to the use of biomarker methods for estimating HIV incidence, providing that good care is taken with sample collection and analysis. The more serious problems lie in: (i) accurately estimating the false-recent rate for every population where a particular method is being used: (ii) the development of bio-marker methods with markedly lower false-recent rates than those typical of the BED.

## References

[1] Parekh BS, Kennedy MS, Dobbs T, Pau CP, Byers R, et al (2002) Quantitative detection of increasing HIV type 1 antibodies after seroconversion: a simple assay for detecting recent HIV infection and estimating incidence. AIDS Res Hum Retroviruses 18: 295–307. 11860677 10.1089/088922202753472874

[2] Hargrove JW, Humphrey JH, Mutasa K, Parekh BS, McDougal JS, et al (2008) Improved HIV-1 incidence estimates using the BED capture enzyme immunoassay. AIDS 22: 511–518. 18301064 10.1097/QAD.0b013e3282f2a960

[3] Brookmeyer R (2009) Should biomarker estimates of HIV incidence be adjusted? AIDS 23: 485–491. 19165087 10.1097/QAD.0b013e3283269e28

[4] Brookmeyer R (2009) Response to correspondence on ‘Should biomarker estimates of HIV incidence be adjusted. AIDS 23: 2066–2068. 10.1097/QAD.0b013e3283269e28 19165087

[5] Hargrove J, van Schalkwyk C, Eastwood H (2012) BED Estimates of HIV Incidence: Resolving the Differences, Making Things Simpler. PLoS One 7: e29736. 22235334 10.1371/journal.pone.0029736 PMC3250478

[6] Kassanjee R, McWalter TA, Bärnighausen T, Welte A (2012) A New General Biomarker-Based Incidence Estimator. Epidemiology 23: 721–728. 22627902 10.1097/EDE.0b013e3182576c07 PMC3500970

[7] Humphrey JH, Iliff PJ, Marinda ET, Mutasa K, Moulton LH, et al (2006) Effects of a single large dose of vitamin A, given during the postpartum period to HIV-positive women and their infants, on child HIV infection, HIV-free survival, and mortality. J Infect Dis 193: 860–871. 16479521 10.1086/500366

[8] Humphrey JH, Hargrove JW, Malaba LC, Iliff PJ, Moulton LH, et al (2006) HIV incidence among post-partum women in Zimbabwe: risk factors and the effect of vitamin A supplementation. AIDS 20: 1437–1446. 16791019 10.1097/01.aids.0000233578.72091.09

[9] Turnbull BW (1976) The Empirical Distribution Function with Arbitrarily Grouped, Censored and Truncated Data. Journal of the Royal Statistical Society Series B (Methodological) 38: 290–295.

[10] Sweeting MJ, De Angelis D, Parry J, Suligoi B (2010) Estimating the distribution of the window period for recent HIV infections: a comparison of statistical methods. Stat Med 29: 3194–3202. 21170913 10.1002/sim.3941 PMC3470924

[11] Team RDC (2011) R: A language and environment for statistical computing. R Foundation for Statistical Computing, Vienna, Austria ISBN 3–900051–07–0.

[12] Lunn DJ, Thomas A, Best N, Spiegelhalter D (2000) WinBUGS – a Bayesian modelling framework: concepts, structure, and extensibility. Statistics and Computing 10: 325–337.

